# Commercial and business aspects of alpha radioligand therapeutics

**DOI:** 10.3389/fmed.2022.1070497

**Published:** 2023-02-02

**Authors:** Emanuele Ostuni, Martin R. G. Taylor

**Affiliations:** ^1^ARTbio Inc., Cambridge, MA, United States; ^2^F-Prime Capital, London, United Kingdom

**Keywords:** ART, distributed manufacturing, capacity, logistics, isotopes

## Abstract

Radioligand therapy (RLT) is gaining traction as a safe and effective targeted approach for the treatment of many cancer types, reflected by a substantial and growing commercial market (valued at $7.78 billion in 2021, with a projected value of $13.07 billion by 2030). Beta-emitting RLTs have a long history of clinical success dating back to the approval of Zevalin and Bexxar in the early 2000s, later followed by Lutathera and Pluvicto. Alpha radioligand therapeutics (ARTs) offer the potential for even greater success. Driven by ground-breaking clinical results in early trials, improved isotope availability, and better understanding of isotope and disease characteristics, the global market for alpha emitters was estimated at $672.3 million for the year 2020, with projected growth to $5.2 billion by 2027. New company formations, promising clinical trial data, and progression for many radioligand therapy products, as well as an inflow of investor capital, are contributing to this expanding field. Future growth will be fueled by further efficacy and safety data from ART clinical trials and real-world results, but challenges remain. Radionuclide supply, manufacturing, and distribution are key obstacles for growth of the field. New models of delivery are needed, along with cross-disciplinary training of specialized practitioners, to ensure patient access and avoid challenges faced by early RLT candidates such as Zevalin and Bexxar. Understanding of the history of radiation medicine is critical to inform what may be important to the success of ART–most past projections were inaccurate and it is important to analyze the reasons for this. Practical considerations in how radiation medicine is delivered and administered are important to understand in order to inform future approaches.

## Introduction

Alpha radioligand therapeutics (ARTs) have been gaining increasing attention as a rapidly advancing experimental modality that holds promise for delivering high doses of lethal radioactivity specifically to cancer cells. The combination of the high energy and short tissue range typical of alpha-emitting isotopes enables effective killing of the targeted tumor while sparing the surrounding normal tissue. ARTs offer the potential to overcome resistance to beta-emitting radioligand therapies, which have already entered the market, or chemotherapy drugs. The promise of alpha has led to growth in new clinical trials and new company formations fueled by risk-tolerant investors.

In this chapter, we explore the history of the targeted radioligand therapy commercial landscape, including the approval and performance of key drug candidates that have shaped the current and future directions of the field. We provide an overview of the current market and its potential, as well as challenges faced in therapeutic and isotope availabilities and barriers for the delivery of ARTs at commercial scale.

## Section 1: Historical context

### The foundations of nuclear medicine and targeted therapies

The origins of radiotherapy start with the discovery of X-rays as the first radiative source by Wilhelm Conrad Röntgen in 1895, who realized their ability to penetrate human flesh to allow photography of higher-density substances such as bone. As diagnostics applications flourished, the ability of X-rays to selectively kill rapidly dividing cells did not go unnoticed. The clinical usefulness of radiation to treat cancer was observed in 1896, when Grubbe used X-rays from an improvised X-ray tube to treat patients with breast cancer and later lymphoma (1, 2).

However, it was Marie Skłodowska Curie who laid the real foundations for ART, and nuclear medicine in general, with the discovery of polonium and radium in 1898. Later, in 1902, Marie and Pierre Curie identified and purified radium-226 in the form of radioactive mineral salts isolated from radioactive pitchblende in their laboratory in Paris. In the following year, they shared the 1903 Nobel Prize in Chemistry with fellow scientist A. Henri Becquerel for their ground-breaking investigations of radioactivity, following the first observations that tumor-forming cells were destroyed faster than healthy cells when exposed to alpha-emitting radium-226 (3).

Early in its development, X-ray based radiation medicine struggled against its limits: directionality and localization, collateral damage. Therefore, many cancer physicians instead turned their attention to surgical techniques and other approaches (4). Nevertheless, ongoing innovation in external beam radiation and brachytherapy has been a hugely important development in cancer treatment, discussed in detail below.

While the physics and applications of radiation were being investigated, researchers remained intrigued by the concept of a molecular “magic bullet”–a term coined by Paul Erlich–to selectively deplete cancer cells while sparing healthy tissue. An array of approaches to achieve this effect has since been deployed in oncology, building on huge advances in cell and molecular biology over the past 50 years. This culminated many years later with the exciting possibility of being able to selectively direct a radioactive warhead to a target highly expressed uniquely on a cancer cell to engender selective cell killing.

### Modern day applications

Great progress has been achieved through radiation-centric approaches in the fields of diagnostics, nuclear medicine, and targeted therapies. Millions of lives have been saved as a result of faster and accurate diagnosis and treatment of injuries and diseases that would not have been possible without nuclear medicine, with significantly improved delivery of care. The medical X-ray market was estimated to be worth $12.4 billion in 2020 (5), while the global radiology market was valued at $26.6 billion in 2021 and is expected to reach $43.0 billion by 2029 (6).

The targeted therapeutics market has also grown substantially, valued at $67.7 billion in 2020 and projected to reach $87 billion by 2030 (7), with multiple targeted agents now approved for diseases such as cancer.

The use of nuclear medicine in oncology has also grown significantly: approximately 50% of all cancer patients receive radiation therapy during their course of illness, with two modern day applications of radiotherapy–external beam radiation therapy (EBRT) and brachytherapy–making up the bulk (8). One analysis of the US Surveillance, Epidemiology, and End Results (SEER) database estimated that 3.05 million cancer survivors were treated with either brachytherapy or EBRT in a single year, accounting for 29% of all cancer survivors that year–with breast (40%) and prostate cancer (23%) patients comprising the majority of radiation-treated survivors (9).

### External beam radiation therapy and brachytherapy–Lessons learned

EBRT used to deliver high-energy X-ray or electron beams to a patient’s tumor.

Modern-day EBRT has proven to be hugely successful for its target indications. Men with high-risk localized prostate cancer treated with EBRT have a cure rate of around 91% (10): 10-years overall survival is above 80% (11), leading to commercial success (12). The global radiation oncology market is valued at $6.8 billion in 2020: EBRT dominated the field with a 79.3% revenue share in 2020, with expectations that it will continue to expand to reach revenue of $11.6 billion by 2030 (13).

Brachytherapy comes in the form of seeds, ribbons or wires placed within the body, in or near the tumor site. High-dose-rate brachytherapy temporarily introduces iridium isotopes close to the tumor site to deliver a higher dose of radiation over a shorter period of time and overcomes limitations of early brachytherapy approaches (14). Evidence-based medicine indicates that brachytherapy may be superior to EBRT in terms of efficacy and safety in several patient groups (15, 16). Survival rates are remarkable: 17-years survival of 97% in prostate cancer (17); 79.4% 3-years survival in cervical cancer patients (18).

These results supported the global brachytherapy market valuation of $788.5 million in 2020 with an expected compounded annual growth rate (CAGR) of 7.1% from 2021 to 2028 (13).

Despite the evidence supporting brachytherapy as an effective treatment modality for a wide range of malignancies, its use to treat patients with localized prostate cancer in the US and Europe saw a steady decline in recent years (19); the percentage of prostate cancer patients receiving brachytherapy dropped from 17% in 2002 to 8% in 2010 (20, 21).

**FIGURE 1:**
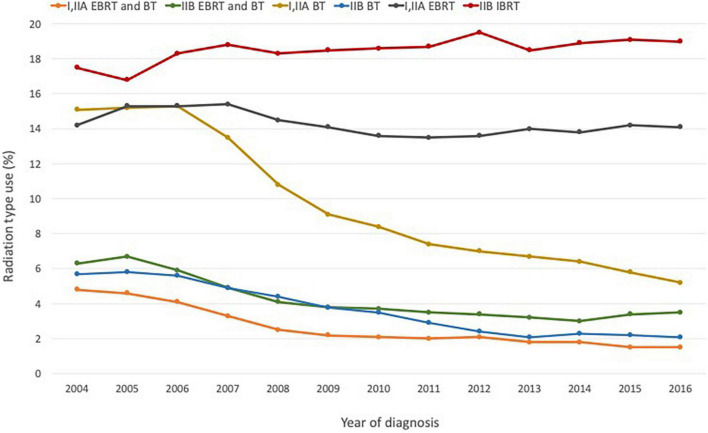
Radiation modality by stage and diagnosis year for prostate cancer based on NCDB data for the period 2004–2016. Figure adapted with permission from (23), ACS.

A significant reason for this decline is the development of more technologically sophisticated treatments, including robot-assisted surgery and proton therapy, as well as more advanced forms of non-invasive EBRT such as IMRT and SBRT (20–26; Figure 1). Falling rates of brachytherapy administration in the US in favor of EBRT have also been attributed in part to financial considerations–a shift partly facilitated by hospital reimbursement policies that favor newer approaches. Brachytherapy is more labor- and cost-intensive for hospitals–some studies have shown that the total cost and staff time devoted to brachytherapy are double those of EBRT (27). In addition, the reimbursement levels set for EBRT are nearly double those for brachytherapy (22, 23).

### The first targeted radio-immunotherapies

Although EBRT and brachytherapy remain two of the most efficient tools for eliminating isolated and discrete cancer, their application in treatment of more advanced and systemic disease is limited. In parallel to their development, nuclear medicine pioneers such as Saul Hertz experimented with the therapeutic applications of metabolically targeted radionuclides, such as iodine-131 in thyroid cancer. Further major advances in this area occurred after the development of peptide receptor radionuclide therapy (PRRT) in the late 1980s by Mark Kaminski, Richard Wahl and colleagues at the University of Michigan (28, 29). In this approach, an engineered peptide (or antibody) aimed at a specific marker found in abundance on cancer cells would carry a radioactive atom capable of delivering a lethal dose of radiation to the tumor–creating a magic bullet against cancer.

Further developments in antibody conjugate technologies led to the launch of monocloncal antibody (mAb)-targeted radiotherapeutics in the early 2000s. Zevalin (yttrium-90-labeled anti-CD20 mAb) and its competitor Bexxar (iodine-131-labeled anti-CD20 mAb) were the first pioneers to appear on the market within this new class, approved for treatment-resistant slow-growing lymphoma.

### Zevalin

^90^Y-ibritumomab tiuxetan (later marketed as Zevalin) is a radioactive drug product comprised of the beta-emitting isotope yttrium-90 linked to the mAb ibritumomab in conjunction with the chelator tiuxetan, and was designed to target the already validated cancer protein marker CD20 (30).

Developed by IDEC Pharmaceuticals, now part of Biogen Idec, ^90^Y-ibritumomab tiuxetan was the first radioimmunotherapy drug approved by the FDA to treat cancer. The drug had a superior response rate in patients who did not respond to rituximab (marketed as Rituxan by Genentech/Biogen Idec) (31–33).

^90^Y-ibritumomab tiuxetan was approved by the FDA (2002) and EMA (2004) for treatment of patients with relapsed or refractory low-grade, follicular non-Hodgkin’s lymphoma, including patients who were refractory to rituximab, and as consolidation therapy in follicular non-Hodgkin’s lymphoma in patients who achieved a partial or complete response to first-line chemotherapy.

**FIGURE 2:**
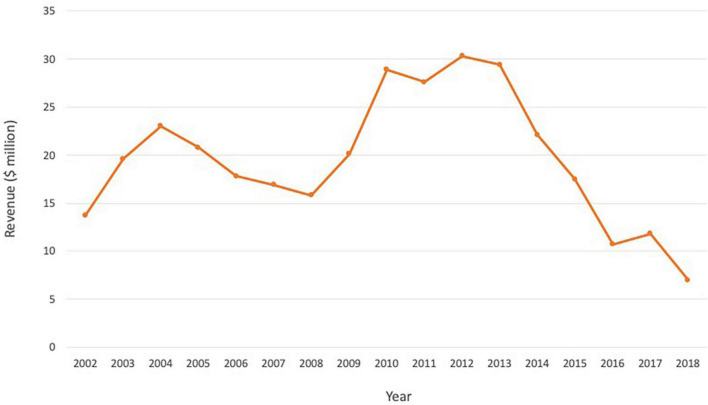
Annual revenue for Zevalin over the period 2002–2018, reflecting a steady decline and failure to meet forecasts. Source: Biogen and Spectrum financial reports.

When Zevalin first came onto the market, Wall Street analysts had projected that sales would reach $100 million in 2003 (34). Merrill Lynch predicted it could eventually hit $500 million in sales, equivalent to approximately 20,000 doses a year (34). Despite the efficacy, better response rate compared to Rituxan, and an acceptable safety profile, Zevalin failed to meet forecasts (Figure 2). The launch was slow and it reached $15–30 million annually in the first decade, before undergoing a steady decline in sales from 2013 (Biogen and Spectrum financial reports). Issues cited with the slow uptake include high price, complicated prescribing, administration and monitoring process, and preference for familiar tools and processes and non-radioactive competitors amongst physicians (Table 1). The drug was divested by Spectrum Pharmaceuticals but is currently marked by Aurobindo Pharma Ltd., to treat non-Hodgkin’s lymphoma in Europe, and by its subsidiary Acrotech Biopharma L.L.C. in the US (35).

**TABLE 1:** Reasons cited for the commercial challenges of Zevalin and Bexxar, highlighting market-driven forces that contributed to declining sales and discontinuation of the drugs.

	Zevalin	Bexxar
Preference for familiar tools and processes amongst physicians	X	X
Complicated prescribing, administration, and monitoring process	X	X
Complicated referral/referral outside of doctors’ offices	X	X
Complex dosimetry requirements	X	X
Unclear data around long-term benefit/outcomes	X	X
Potential toxicities	X	X
High price/costs	X	X
Reimbursement challenges	X	X
Clinical trial strategy challenges/delays with FDA		X
Manufacturing and supply-chain challenges	X	X
Public fears about radiation risks	X	X

Note that Zevalin is still marketed for use in Europe.

### Bexxar

^131^I-tositumomab (later marketed as Bexxar) was a radio-immunotherapeutic composed of the mAb tositumomab covalently bound to the radioisotope iodine-131. The compound was also targeted at the CD20 antigen and delivered a powerful local dose of gamma and beta radiation.

The drug was developed in the late 1990s by Coulter Pharmaceutical and acquired in 2000 by Corixa (36), who attracted significant investment for the manufacturing and marketing of the drug. Along with support from big pharma partner Glaxo Smith Kline (GSK), ^131^I-tositumomab had promising clinical trial data–its pivotal study enrolled 40 patients with non-Hodgkin’s lymphoma with no treatment options following failed attempts with rituximab and several rounds of chemotherapy. Sixty-three percent of patients experienced significant tumor shrinkage with ^131^I-tositumomab and the benefit lasted more than 2 years (median 25 months), with 29% percent of patients entering complete remission. These results were supported by four additional single-arm studies in which overall response rates ranged from 47 to 64% with median response durations of 13–16 months (37).

The drug was granted orphan drug designation in 1994, and fast-track designation was added in 1998. ^131^I-tositumomab was first approved by the FDA and EMA in 2003 for patients refractory to rituximab or that had relapsed following chemotherapy; in 2004, the indication was expanded to include patients who had not been treated with rituximab. Approval was delayed in the US, however, by a series of FDA requests for information, and was granted 4 years after the new drug application was filed in June 1999. During those 4 years, the competing combination of Rituxan and chemotherapy established itself as the standard of care in non-Hodgkin’s lymphoma.

Forecasts of Bexxar’s market potential were high on the basis of an earlier US launch. In the year 2000, Data monitor estimated that Bexxar sales would reach $350 million by 2005; in February 2001, ABN Amro Predicted launch in 2001 and sales of $25 million, rising to $70 million in 2003. Bexxar sales failed to meet expectations following the delay by the FDA. First-quarter 2004 sales were $1.3 million, rising to just $2.2 million in the second quarter of 2004 (36).

Corixa, despite having remarkable clinical trial data, struggled to turn Bexxar into a commercial success, and was acquired by GSK in 2005 for $300 million. Bexxar usage peaked in 2006, and sales decreased by 30% annually thereafter. In 2012, only 75 patients received Bexxar (38). On 20 February 2014, GSK announced that the manufacture of Bexxar would be voluntarily discontinued, due a projected decline in sales and the availability of alternative treatments. Issues cited more widely included clinical trial strategy and issues with the FDA, complicated patient referral process, supply chain issues, reimbursement, and emergence of non-radioactive competitors. Safety concerns may also have contributed to the drug’s dwindling use, following a 2011 trial suspension for a study comparing the use of ^131^I-tositumomab and rituximab in addition to chemotherapy among patients with newly diagnosed follicular lymphoma, an indication for which Bexxar had not received approval. Survival was worse in ^131^I-tositumomab arms of the study, and although not statistically significant, the results highlighted potential harms such as severe allergic reactions at the time of infusion and cytopenia (38).

### Market-driven challenges of Zevalin and Bexxar

Zevalin and Bexxar, as first-in-class targeted radiotherapeutics, shared some common commercial penetration issues (Table 1). Both drugs faced competition from Genentech and Biogen Idec’s blockbuster drug Rituxan, which was the leading treatment at the time, and were considered expensive at around $25,000 per treatment. However, as one dose is usually enough, the cost of the drugs was actually similar to a full 4-months regimen of chemotherapy and Rituxan.

The radioactivity of the treatments made some oncologists worry that it might prevent them from giving other treatments later. Prescribing the drugs also requires oncologists to coordinate care with the hospitals that administer it–to get either drug, patients first receive a low-radiation diagnostic dose, then imaging scans, then a high-radiation therapeutic dose, which comes a week after the first dose. Other more familiar and thoroughly tested drugs were also preferred as first-line treatment, leading physicians to prescribe such drugs even when Zevalin and Bexxar might have worked better. Financial incentives were also at play–as Zevalin and Bexxar were radioactive, they were administered in hospitals by nuclear medicine experts following a referral by hematologists, who were likely to lose revenue in some markets. As a result, referral rates were lower than they could have been based on the product labels.

This led to the use of Zevalin and Bexxar as last resort treatments only. In 2007, it was estimated that fewer than 10% of lymphoma patients who were candidates for Zevalin and Bexxar ever received the therapies (39). Despite the potential and clinical data of the two drugs, the positive sales forecasts, and non-Hodgkin’s lymphoma being a common cancer in Europe and the US that accounts for around 4% of all cases (40), the commercial challenges reflect the market-driven forces and the lack of coordination among physicians that can distort medical decisions.

### The arrival of alpha

While beta-emitters Zevalin and Bexxar traversed along their respective journeys, the development of targeted radionuclide therapies using different alpha-emitters was also in progress. The first alpha emitter to appear on the market was metabolically targeted, analogous to ^131^I for thyroid cancer.

### Xofigo

^223^Ra-dichloride (later marketed as Xofigo) was the first alpha-emitter to enter the market. Once injected into the blood, its active moiety radium-223 mimics calcium and selectively targets bone due to natural tropism, with high specificity for areas of bone metastases.

First developed by Algeta and later by Bayer following a $2.9 billion acquisition, ^223^Ra-dichloride was designed to treat metastatic castration-resistant prostate cancer (mCRPC). In its pivotal ALSYMPCA Phase III trial, the compound resulted in a 30% reduction in the risk of death compared with placebo, and extended patient lives by a median of 14 months compared to 11.2 months (41, 42).

Use of ^223^Ra-dichloride was approved by the FDA in 2013 for mCRPC patients with symptomatic bone metastases. This was more than 3 months ahead of schedule due to the FDA’s priority review program, with the trial ending early due to the drug’s strong performance–reasons cited included the drug’s precise targeting and strong risk–benefit profile. Approval was also received from the EMA in 2018.

**FIGURE 3:**
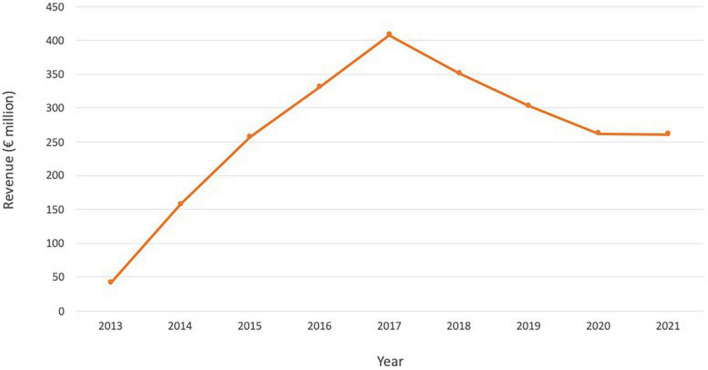
Annual revenue for Xofigo over the period 2013–2021. Source: Bayer annual reports.

Xofigo had very high commercial promise due to its high efficacy and targeting specificity, and its potential to treat late-stage prostate cancer patients with few other options. It was heralded as one of Bayer’s “Big Five” crucial new drugs, and analysts estimated that annual sales could peak at around $1.5 billion by 2020 (43). However, although Xofigo fared significantly better than Zevalin and Bexxar, with sales reaching $300–400 million annually at its peak (Figure 3), it also faced challenges. Firstly, the prostate cancer market evolved rapidly with many non-radioactive competitors. Secondly, in 2017, safety concerns arose when the Phase III Era-223 clinical trial for use of ^223^Ra-dichloride in combination with abiraterone acetate (Johnson and Johnson’s Zytiga) in mCRPC patients pre-chemo was terminated early. In the trial, the combination caused more fractures and deaths than abiraterone acetate alone (44). The resulting negative perceptions of the drug, the challenges to extend its use to earlier stages of prostate cancer, and the difficulties in combining with other emerging important prostate cancer medicines, made Xofigo subject to the increasing competition provided by new therapies. Xofigo may face additional commercial threats from the recently approved targeted radioligand therapy Lu-177-PSMA-617 (Pluvicto), which has the potential for utility in a broader population of metastatic prostate cancer patients; unlike Xofigo, Lu-177-PSMA-617 use is not restricted to patients with metastases predominantly in bone.

Following the failed trial and fast-changing nature of the prostate cancer market, analyst sales estimates fell. Xofigo revenues were no longer expected to breach $500 million in 2017, more than 4 years from launch (43). In 2018, Xofigo suffered a double-digit sales decline that continued for several years (Figure 3), exacerbated by COVID-19 restrictions (45). Despite the challenges, Xofigo remains an approved therapy for the treatment of prostate cancer and ^223^Ra-chloride is undergoing further evaluation in several ongoing trials; the commercial performance of Xofigo has far exceeded those of the beta-emitters Zevalin and Bexxar (42).

## Section 2: Present

### Radioligand therapeutics come to life

Since the approvals of Zevalin, Bexxar and Xofigo, momentum has continued in the field. Promising proof-of-concept signals from small compassionate-use case series, investigator-led clinical trials, and improvements in tumor-targeting technologies resulted in more refined and optimized targeted RLTs. The next pivotal step in the evolution of the field came in the form of two major commercial transactions, Novartis’ acquisitions of Advanced Accelerator Applications (AAA) and Endocyte in 2018.

### Movement in the RLT field: Novartis acquisitions of advanced accelerator applications and endocyte

In January 2018, Novartis announced the completion of its $3.9 billion ($41 per share) acquisition of radiopharmaceutical specialist AAA and its RLT candidate ^177^Lu-DOTATATE (later named Lutathera).

^177^Lu-DOTATATE, which combines the beta-emitting radionuclide lutetium-177 with the somatostatin analogue DOTATATE to target somatostatin receptors (SSTRs) on tumor cells, was the first radiopharmaceutical on the market for PRRT (46). The drug gained rapid approval for clinical use following ground-breaking clinical data–in the NETTER-1 Phase III study of 229 patients with inoperable SSTR-positive advanced midgut neuroendocrine tumors, ^177^Lu-DOTATATE increased progression-free survival (65% versus 11% survival at 20 months) and response rate (18% versus 3%) compared with high-dose octreotide LAR (Sandostatin LAR Depot) (46–48). These results led to authorization by the EMA (2017) and the FDA (2018) for the treatment of SSTR-positive gastroenteropancreatic neuroendocrine tumors. The drug has also show potential in off-label use in other neuroendocrine tumors (e.g., bronchial) in both the US and Europe.

In December of the same year, Novartis announced completion of its $2.1 billion takeover of Endocyte and its lead asset ^177^Lu-PSMA-617 (later named Pluvicto). ^177^Lu-PSMA-617 was a RLT candidate in development against prostate-specific membrane antigen (PSMA)-positive mCRPC. Upon completion of the Phase III VISION trial, it was shown that ^177^Lu-PSMA-617 with standard of care reduced risk of death by 38% compared to standard of care alone and increased progression-free survival (8.7 months versus 3.4 months in the control group) and overall survival (15.3 versus 11.3 months) (49, 50). ^177^Lu-PSMA-617 became the first RLT to be approved by the FDA and EMA for mCRPC, receiving authorization from both agencies in 2022 alongside ^68^Ga gozetotid (Locametz)–a PSMA-targeted positron emission tomography imaging tracer that is used to identify patients suitable for treatment with the radioligand.

Novartis have initiated additional early stage development programs for ^177^Lu-PSMA-617 in earlier lines of prostate cancer therapy, with two other Phase III studies for mCRPC now ongoing. If successful, these trials could significantly increase the patient pool eligible for ^177^Lu-PSMA-617.

### A commercial success story, so far

**FIGURE 4:**
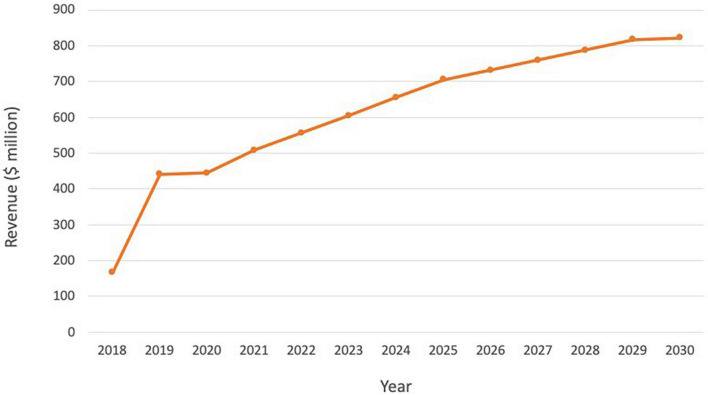
Lutathera sales and projected sales for the period 2018–2030. Source: (51).

Following the ground-breaking clinical data and approvals in US and Europe, and despite its indication for a rare cancer type, Lutathera brought in sales of $445 million in 2020, reaching over 5,000 patients (Table 2). Sales rose to $475 million in 2021 and continued to grow in all regions with approximately 450 centers now actively treating patients globally. As Lutathera becomes accessible to more hospitals and clinics, the number of patients qualifying for the treatment is projected to increase. Analysts predict peak sales of Lutathera could exceed $800 million (Figure 4; 51).

**TABLE 2:** Lutathera revenue and estimated number of doses and treatments for the period 2018–2021.

Years	Lutathera revenue ($ million)	ASP per dose ($)	Implied number of Lutathera doses	Implied number of treatments (4 doses per treatment)
2018	167	20,000	8,350	2,088
2019	441	20,000	22,050	5,513
2020	445	20,000	22,250	5,563
2021	475	20,000	23,750	5,938

Source: (93), Novartis annual report 2021.

**FIGURE 5:**
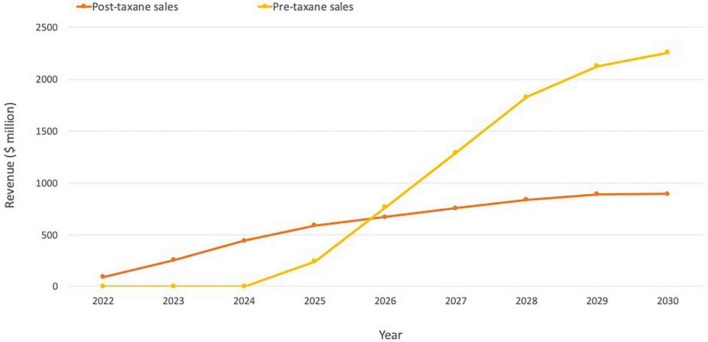
Pluvicto sales projections. Projections include estimates for both pre- and post-taxane markets assuming ∼20% penetration in the US and ∼15% elsewhere. If Pluvicto is approved for the pre-taxane market, it is estimated that this would lead to an additional ∼$2 billion on top of current projections for the post-taxane market. Based on estimates from (51).

Although too early to review longer-term revenue data for Pluvicto, Novartis reported initial sales of $10 million for Q2 2022 (52). Evaluate Vantage recently projected Pluvicto’s 2026 sales of $851 million, while analysts at Jefferies have previously predicted that sales could reach $600 million in the current indication, with additional upside from further approvals (including the pre-chemotherapy setting in mCRPC and treatment-naive metastatic hormone-sensitive prostate cancer patients). The two additional trials in progress are expected to drive a 2–3x increase in currently modeled sales if successful, indicating the blockbuster potential of Pluvicto (Figure 5; 51).

In 2022, Novartis forecast annual sales up to or exceeding $1 billion for both Pluvicto and Lutathera, which together represent a major opportunity for Novartis in nuclear medicine (53). The company has also continued to increase its exposure to radiopharmaceuticals–for example by participation in the Series A financing of Aktis Oncology and the in-licensing of a other targeting agents from SOFIE Biosciences.

### Growth of new candidates and companies for RLT

The acquisition of AAA and Endocyte by Novartis triggered significant and growing interest and expectations for RLTs. The subsequent approvals and early robust market uptakes of the two lutetium-based drugs coupled with lofty future projections suggest better market readiness for RLTs than at the time of the launches of Zevalin and Bexxar two decades ago. This commercial success has in turn sparked the interest of investors and other large pharmaceutical companies looking to address unmet needs in cancer.

Several developments facilitated further expansion of the RLT concept for oncology. These included improved drug targeting; the increased availability of ^177^Lu and growing investment in production of alpha emitters; advances in new processes for efficient manufacturing of RLTs and increasing production capacity; and compelling clinical data. This progress transformed the dynamic, fueling a new flow of investor capital into these technologies and increasing mergers and acquisition (M&A) activity (28). As a result, momentum has continued to build in the nuclear medicine field, with the potential to elevate the profile of the entire sector. If the industry is able to effectively manage historical challenges, there is significant opportunity for a new and promising wave of RLTs to significantly change oncology treatment paradigms–particularly if alpha emitters are effectively utilized.

### Market reception for public and private companies

With this momentum, new company formation has grown since 2018, and pharma giants such as Bayer and Novartis continue to build early stage pipelines that expand into other targets and radioisotopes–with increasing focus on alpha-emitters.

Hard data and future potential attracted significant capital. For instance, prior to the Novartis acquisition, after the disclosure of the 79% reduction in the risk of disease progression or death for patients with SSRT-positive neuroendocrine tumors following treatment with ^177^Lu-DOTATATE in the NETTER-1 Phase III trial, AAA raised more than $75 million in an oversubscribed IPO in 2015. Investors again showed their support in October 2016, when AAA raised more than $150 million in a follow-on offering (28).

Private companies have also experienced positive market reception. Analysis indicates that at least 11 companies working in the ART space have raised significant amounts of capital during the period 2019–2022. We estimate the amount raised by those companies totalling close to $1.2 billion, although this estimate is not exhaustive given the private nature of some of this information. Much of the focus of this new investment has been on targeted alpha approaches as investors seek out opportunities with differentiated clinical efficacy potential. Investment has also continued into companies pursuing beta-based approaches which have a different risk profile given the existence of two approved products and a more established supply chain.

### Current state of the market

Promising clinical trial data, the inflow of investor capital, and M&A activity are contributing to an expanding radionucleotide field.

The overall global nuclear medicine market size expected to reach $24.4 billion by 2030 at a CAGR of 13.0% from 2022 to 2030 (54). Meanwhile, the global market for radioligand therapy is projected to reach $13.07 billion by 2030 (55). This is a reflection of increased public and private funding and clinical progression for many RLT products between 2018 and 2022, as well as increasing cancer prevalence. Other opportunities and drivers for further growth in the RLT market include the aging population, increased awareness and understanding of radiotherapy isotopes, product innovation and development, and improvements to isotope production and infrastructure for clinical use. Increasing use of radiopharmaceuticals by physicians and rising per capita health care expenditure will also boost the market’s growth.

Beta-emitting isotopes currently dominate research efforts, as they have done since the inception of RLT (56). In September 2021, of 161 ongoing registered radionuclide therapy clinical trials, 133 were focused on beta-emitters and 28 on alpha-emitters (57). This has been driven mostly by the availability of isotopes such as lutetium and the market is expected to evolve to reflect a shift to alpha emitter therapeutics. The global market for alpha emitters was estimated at $672.3 million for the year 2020, with projections of $5.2 billion by 2027, indicating a CAGR of 34.1% over the period 2020–2027 (58). In comparison, beta emitters were projected to exhibit a CAGR of only 13.7% (56).

Despite the advances in RLT and the positive outlook of the projected commercial landscape, challenges in the commercial penetration and uptake remain. Primarily, radionuclide supply, manufacturing and distribution, in particular for alpha-emitting radionuclides, are key obstacles for growth of the field. Effective delivery of RLT requires carefully orchestrated manufacturing, transport and preparation of radiopharmaceuticals, and necessitates dedicated infrastructure and mechanisms for waste disposal. The existing model for manufacturing, transporting and preparing radioligand therapy is suitable for administering the therapy to a limited number of people per week, and so there is a need to develop different models for larger patient populations. These models of delivery will need to account for differences in radiopharmaceuticals, eligibility assessment techniques and number of treatment cycles (to be explored further in Section “Exploring models for the delivery of ART”) (59, 60).

Additional challenges include the failure by physicians to adopt and rigorously evaluate this treatment modality, which may be explained in part by the multidisciplinary nature of the treatment and financial incentive challenges, as experienced by Zevalin and Bexxar (59). Public perception and fear of radioactivity, as well as the perceived complexity of the treatment, may also be a difficulty, but one that can be overcome with better communication of risk–benefit profiles and increasing positive data around side effects and effectiveness.

## Section 3: The future is alpha

Radioligand therapy (RLT) is a growing market despite the challenges faced. Assuming that the early ground-breaking results obtained with ART continue to be borne out in rigorous clinical trials, the growth of ART is also likely to accelerate over the use of EBRT.

### Benefits of alpha

Alpha particles are helium nuclei that are emitted from the nucleus of a radioactive atom. The amount of energy deposited per path length traveled (linear energy transfer or LET) is approximately 1,500 times greater than beta particles, leading to substantially more damage along the path of travel (59, 61, 62).

Depending on their emission energy, alpha particles can travel 50–100 μm in tissue. The combination of high energy and a short tissue range ensures the deposition of a large amount of energy within a short radius, leading to the effective killing of the targeted tumor with sparing of the surrounding normal tissue. This occurs due to direct DNA damage from alpha particle collisions with DNA, leading to severe DNA double-strand breaks, which are difficult to repair and trigger cell death. This is a key advantage of alpha-emitters as double-strand breaks are harder for a cell to survive than the single-stranded breaks induced by beta radiation (59, 61, 62).

### Differences among alphas

For radionuclides to be used effectively over time, commensurable with their half-life period, it is necessary to produce and isolate them, perform synthesis with the targeting molecule, and execute control of key parameters such as the absence of long-lived and/or toxic daughters (63–67). Each of these requirements is explored in more detail below.

#### Half-life

A shorter half-life means the radioisotopes must be isolated closer to the time and site of treatment, whereas a longer half-life means the radioisotope can be produced in a specialized, central location and subsequently delivered to hospitals and clinics, provided that the daughters can be stable in the complexes during delivery. The 9.92-day half-life of actinium-225 (^225^Ac) is suitable from this perspective, but the poses potential toxicity risks stemming from mother radionuclide recoil caused by the energy from four successive alpha emissions in its decay cascade. In addition, care must be taken to ensure the quality of the product is not compromised by prolonged storage periods, which can occur due to radiolysis from the targeting ligand–these characteristics may limit the deployment of ^225^Ac therapeutics. Lead-212 (^212^Pb), with a shorter but still manageable half-life of 10.64 h, decays to bismuth-212 (^212^Bi) (*T*_1/2_ = 1 h) and is used as a means to deliver ^212^Bi without being constrained by its shorter half-life. This allows for delivery of up to 10 times more dose per unit of administered activity and provides the possibility for the synthesis of complex radiopharmaceuticals with minimum loss of radioactivity during preparation (66).

#### Ability to complex

For a radiopharmaceutical to be used successfully, it must manifest sufficient stability *in vivo* to retain its targeting properties, and in the case of metal isotopes an appropriate chelator needs to be identified that matches the physical properties of the isotope to link the isotopes to targeting ligands (68, 69). With target in mind, the half-life of the isotope should also be compatible with the characteristics and half-life of the vector molecule (64, 65). Astatine-211 (^211^At) (*T*_1/2_ = 7.2 h) and ^212^Pb (*T*_1/2_ = 10.64 h) exhibit favorable characteristics in this regard, with half-lives that are suitable to the kinetics of small peptides and small molecules that require short periods to reach an optimal tumor-to-blood dose ratio, as well as high decay efficiencies and stability to reduce toxicity (61, 64). Isotopes with longer half-lives are often complexed with long-lived antibodies: while the targeting is adequate, the long circulation times of antibodies may increase the risk of non-specific toxicity and off-target effects, e.g., toxicity to the bone marrow.

#### Toxicity

Many isotopes emit alpha particles but some leave behind toxic by-products or decay before they reach a cell. Issues arising when using ^225^Ac for therapy, for example, as mentioned above, include unwanted toxicity from recoiled daughter radionuclides without a targeting ligand (70). Upon the emission of an alpha particle, the radioactive daughter nuclides experience a recoil energy of about 100–200 keV, which is sufficient to allow the daughter nuclide to break free from the targeting agent. Further, the different chemical properties of the daughter radionuclide can make re-association with the chelator unlikely. These “free,” untargeted daughter nuclides could be a source of dose-limiting toxicity.

When these factors are taken into account, despite that many different alpha-emitting radionuclides have been identified, only a few have desirable characteristics that render them suitable for clinical application (66, 67). Of the alpha-emitting radionuclides that have been identified as suitable for therapeutic use, several candidates have now been complexed to ligands such as PSMA inhibitors for evaluation in preclinical and clinical studies for cancer such as mCRPC (71). Following these early evaluations, four of the most promising isotopes emerging within the ART field are ^225^Ac, ^211^At, ^212^Pb, and thorium-227 (^227^Th)–although ^213^Bi has been used with positive results in select malignancies, we are not aware of large scale commercial efforts with this isotope.

### Isotope availabilities

Medical isotope shortages are a concern globally due to limited source material and challenging production processes. Although many isotopes are produced in nature, extracting a significant amount of purified material demands an accelerator or nuclear reactor and the facilities and expertise to chemically separate out the desired isotope from many others created during production. Other strategies include generators, where a parent isotope decays to the desired radionuclide that is then extracted, and cyclotrons that accelerate and bombard a target using variety of particles, including protons, alpha particles, lithium, and carbon ions.

For the four isotopes identified as most suitable for therapeutic use, the availability and ease of production are therefore a key factor to consider for their use. Below is a state-of-play for each, including current and potential future availability and production methods.

#### Astatine-211

^211^At can be produced at reasonable yield and high radionucleic purity using an alpha-particle beam to bombard natural and widely available bismuth at ∼28 MeV *via* cyclotron irradiation. Despite being a straightforward method of production, the number of accelerators capable of a 28 MeV alpha-beam limits the availability of ^211^At, and current quantities are inadequate for widespread clinical use (72).

#### Lead-212

The main production route of ^212^Pb is through the use of radium-224 (^224^Ra)-based generators from which ^212^Pb is obtained by elution. This does not come without challenges–the generator must be replaced after 1–2 weeks due to the short half-life of ^224^Ra–but it can produce high yields of ^212^Pb (> 90% of expected activity per daily elution) and its daughter ^212^Bi at quantities sufficient for preclinical and clinical use. The US Department of Energy’s Oak Ridge National Laboratory (ORNL) currently produces ^212^Pb using this approach, and some biotechnology companies are also developing their own facilities and methods to produce high-purity ^212^Pb (61). The short half-life of ^212^Pb and the relatively long separation times of the methods above reduced its applicability to date. However, several companies such as ARTBIO recently started to innovate such production processes and made significant process toward scaling up the supply of ^212^Pb through sustainable methods (73). While the specific production and purification methods of ^212^Pb are under development, there is good availability of the potential parent radionuclide ^228^Th, which provides good confidence in the ability of these approaches to ultimately scale to accommodate commercial therapeutic volumes.

#### Actinium-225

^225^Ac has limited availability as it can currently only be extracted by separation from the natural decay of ^229^Th that is obtained from waste stockpiles containing ^233^U (from past reactions for nuclear energy or nuclear weapons purposes). At present, there are two sources of ^225^Ac that have been used in clinical trials, held at ORNL in the US and the Institute for Transuranium Elements (ITU) in Karlsruhe, Germany. Additional sources are also available at the Leypunsky Institute for Physics and Power Engineer (IPPE) in the Russian Federation, South Africa’s iThemba Laboratory for Accelerator Based Sciences and Canada’s TRI-University Meson Facility (TRIUMF)” (74–77, 78, 79) Table 4 lists the overall available capacities of current and future methods (75). Future production methods in development for the production of ^225^Ac include neutron, proton and deuteron irradiation of ^226^Ra targets, and high-energy proton irradiation of ^232^Th targets. Large-scale production of ^225^Ac by cyclotron proton irradiation of ^226^Ra has also shown promise (75).

**TABLE 3:** Overview of current and potential production methods for four key alpha-emitting isotopes.

Isotope	Half-life	Isotope availability	Main production approach	Current and potential production methods	
				Method	Status
^211^At	7.21 h	Very low	Cyclotron	^209^Bi(α,2n)^211^At	Production
				^232^Th(p,x)^211^Rn	Research
				^238^U(p,x)^211^Rn	Research
				^209^Bi(7Li,5n)^211^Rn	Research
				^209^Bi(6Li,4n)^211^Rn	Research
^212^Pb/^212^Bi	10.64/1 h	Scaling	Generator decay	^224^Ra/^212^Pb generator	Production
^225^Ac	9.92 days	Low–growing	Generator decay	^229^Th/^225^Ac generator	Production
				^226^Ra(p,2n)^225^Ac	Research
				^226^Ra(γ,n)^225^Ra	Potential
				^226^Ra(n,2n)^225^Ra	Potential
				^226^Ra(d,3n)^225^Ac	Potential
				^232^Th(p,x)^225^Ac	Research
^227^Th	18.7 days	High	Generator decay	^227^Ac decay	Production
				^235^U decay	Production

Potential routes to increase production for each isotope include: ^211^At: explore production at existing and upcoming facilities and ^221^Rn generator routes; ^212^Pb/^212^Bi: increase production of ^228^Th; ^225^Ac: provide additional stock of ^229^Th, scale up spallation on ^232^Th production and new cyclotron methods; ^227^Th: produce ^227^Ac *via* neutron irradiation of ^226^Ra. Source: (74, 94, 95).

**TABLE 4:** Summary of current and potential future capacity for key ^225^Ac production facilities.

	Production method	Facility	Capabilities	Monthly ^225^Ac production (GBq)
Current sources	^226^Th generator	ORNL	0.704 g of ^229^Th	2.2
		ITU	0.215 g of ^229^Th	1.1
		IPPE	0.704 g of ^229^Th	2.2
Potential sources	^232^Th(p, x)^225^Ac	TRIUMF	500 MeV, 120 μA	11266.5
		BNL	200 MeV, 173 μA	2675.84
		INR	160 MeV, 120 μA	1002.0
		Arronax	70 MeV, 2 × 375 μA	462.1
		LANL	100 MeV, 250 μA	444.0
		iThemba LABS	66 MeV, 250 μA	127.7
Future sources	^226^Ra(p, 2n)^225^Ac	20 MeV, 500 μA cyclotron		3983.1
		15 MeV, 500 μA cyclotron		1157.4
	ISOL	TRIUMF (existing)		0.37
		TRIUMF (potential upgrades)		190.6
	^226^Ra(γ, n)^225^Ra	Medical linac	18 MeV, 26 μA	48.1
		ALTO	50 MeV, 10 μA	55.5
	^226^Ra(n, 2n)^225^Ra	Fast breeder reactor		∼37

Current production levels are listed for current sources, while values for potential sources list estimates of maximum possible production at sample of existing and operational facilities that have dedicated stations for large-scale medical isotope production. This list includes key facilities but is not exhaustive and does not include potential yet currently impractical methods that may be established in the future. However, without knowing details of each institution’s target irradiation facilities, estimates have been based on maximal yield estimates with optimal site assumptions. As a result, practical yields will be lower. For example, while TRIUMF could theoretically produce 11.2 TBq of ^225^Ac per month, 3 TBq of monthly ^225^Ac production is a more practical estimate given the existing target station’s size and cooling capacity. Reproduced from (75).

#### Thorium 227

^227^Th has been commercially available for many years as it can be obtained in clinically meaningful quantities *via* beta-particle decay of ^227^Ac (*T*_1/2_ = 21.8 years). Since it can be produced in virtually unlimited amounts with current technology, ^227^Th has attracted attention as a viable radionuclide for several forms of systemic radionuclide therapy (80, 81). ^227^Th is currently available from ORNL and the Pacific Northwest National Laboratory in the US, the Rosatom State Nuclear Energy Corporation in Russia, and from the pharmaceutical company Bayer (74).

Although production of most alpha-emitting isotopes remains limited, many industry experts assume that capacity will increase as clinical evidence supporting the benefits of ARTs grows over time. In addition, technology development continues in the public and private sectors (59). For example, Table 3 shows current and anticipated production methods for therapeutic alpha-emitter systems. Location of the different facilities will also be important for the scale-up of isotope production for clinical and commercial use, as ART is delivered as a just-in-time therapy. For the widespread treatment of patients in the future, facilities will be needed in each continent to ensure broad access. Growing radioisotopes demand will require sustained efforts from the health and energy sectors to ensure consistent supply and delivery (particularly as there can be additional logistical difficulties in post-production processing and distribution to hospitals) (82).

### The rush to ^225^Ac

^225^Ac has gained much attention as a promising isotope for use in ART, due to its 9.92-day half-life; high LET; manageable chelation and conjugation to targeting molecules such as antibodies and peptides; four net alpha particles emitted per decay for high lethality to target cells; and existing body of early clinical experience (83).

The efficacy of ^225^Ac was demonstrated in early first-in-human patient studies for mCRPC–one of which was conducted under a collaboration between the Joint Research Center in Karlsruhe and University Hospital Heidelberg in 2016 (84). Two patients in highly challenging clinical situations showed a positive response to ^225^Ac-PSMA-617 therapy–both experienced a complete response with prostate-specific antigen decline and no hematologic toxicity, with manageable xerostomia as the only notable side effect (85). While the clinical application of ^225^Ac-PSMA-617 was further developed with the collaboration of JRC and hospitals in Heidelberg, Pretoria and Munich, the remarkable potential of ^225^Ac also gained worldwide interest due to its use in a growing number of studies for patients with late mCRPC (86, 87). Consequently, an increasing number of novel ^225^Ac-labeled compounds are currently under development. We last counted 16 active clinical programs in clinicaltrials.gov and we estimate double that number in pre-clinical stage as many companies do not publish their programs until start of clinical trials.

**FIGURE 6:**
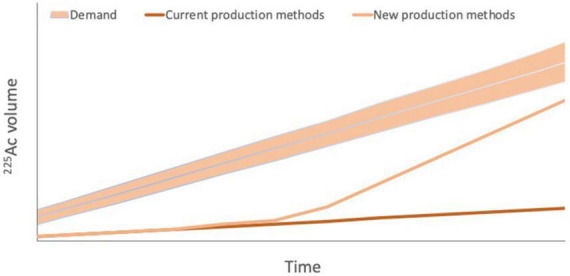
Projected ^225^Ac demand versus current ^225^Ac production *via* ^229^Th production from ^223^U legacy waste and potential future production. Current ^225^Ac production is estimated to be 55–65 GBq per year, which is inadequate even for current demand from researchers. Demand is projected to increase by 200–400 GBq per year for each ^225^Ac-based therapy that is approved for clinical use. Should efforts to develop ^213^Bi-based therapies also increase, ^225^Ac demand may be even higher, highlighting the importance of new production methods to increase ^225^Ac supply to meet increasing demand. However, it should be noted that estimates of both demand and future production capacity vary widely.

However, as noted above, ^225^Ac faces major production challenges due to scarce availability of source material and the infancy of alternative production methods. The total global annual ^225^Ac production volume is approximately 66 GBq, which is inadequate for current and future demand from researchers and for the development of new agents (75; Figure 6). Estimates of current demand for ^225^Ac are less than 185 GBq per year and it is estimated to grow by about 200–400 GBq per year for each ^225^Ac-based therapy that is approved for clinical use. Should efforts to develop ^213^Bi-based therapies also increase, ^225^Ac demand may be even higher (75).

Private and public efforts to increase ^225^Ac supply for medical research and clinical use are ongoing. For example, in 2018, the International Atomic Energy Agency convened a meeting to discuss a global strategy to meet the rising demand for ^225^Ac. The resulting report described potential production routes *via* multiple sources, including proton cyclotrons, linear accelerators, and nuclear waste. The US Department of Energy is also supporting many initiatives to increase production quantities to meet market demand for trials and experimental drugs and is currently leading the Tri-Lab Research Effort to Provide Accelerator-Produced Actinium-225 for Radioimmunotherapy. Private companies such as TerraPower, a leading nuclear innovation company founded by Bill Gates and like-minded visionaries, are also contributing to efforts to increase production. While others are working to ramp up production of ^225^Ac by using a linear accelerator or cyclotron, TerraPower has been working since 2018 to increase the global supply of ^225^Ac from ^229^Th decay, and hopes to harvest the equivalent of 200,000 to 600,000 doses a year (100 times the number of doses currently available globally) from US Department of Energy ^233^U legacy wastes (88).

A delay is expected before production capacity can meet demand. Table 4 provides examples of current and potential sources of ^225^Ac production going forward. Although new production facilities have been set up or are under construction through efforts such as those of the US Department of Energy, new processes for supply expansion have not been fully developed, have only been demonstrated at small scale, or do not currently produce any commercially available quantities. It appears that the shift and rush to ^225^Ac has happened more quickly than with the beta emitter ^177^Lu: in that case, the supply has grown at a rate commensurate with the demand without creating long-term major shortages (89).

There is also significant concern in the sector that the rush to use ^225^Ac before full investigation of the stability of its chelated state and how its long-half life may result in potential toxicity was premature. In addition, the disconnect between supply and demand of ^225^Ac is slowing down academic research and is driving academic and industrial stakeholders to consider alternative isotopes such as ^212^Pb, which has a more favorable decay profile.

## Section 4: Delivery and optimization of ART

### Exploring models for the delivery of ART

There are a number of considerations when selecting an appropriate isotope for use in ART. Once an isotope–molecule combination has been matched to the target disease and its clinical profile, logistics and supply chains must also be built to match. Currently, it appears that several companies may have chosen the isotope first, based on logistics, rather than the approach proposed here. Developers face additional challenges in this space as guidelines and protocols vary between countries, adding complexity to an international delivery solution (82). The scale at which models are implemented may vary, with certain benefits and challenges associated with implementation at a localized or centralized level.

### Localized versus centralized models

A localized model, where manufacturing and administration facilities are co-located, could be beneficial for many reasons. Such a structure may reduce geographical access challenges compared to a centralized model where people are required to travel significant distances, or where isotope choice is limited due to the need to transport therapeutic doses over long distances, even across countries, for treatment. In the early days of RLT, physicians experimented locally in these ways.

A localized model may garner support by physicians as it could provide facilities with their own generators and production stations, improving treatment autonomy and the ease of referrals. Localized models of delivery and care may also alleviate the challenges posed by financial incentives and reimbursement that contributed to the issues experienced by Zevalin and Bexxar.

The regulatory framework for such a model is not well-developed for pharmaceuticals while there is significant experience in radioactive diagnostics: current frameworks would have to be adjusted while the purveyors of such models may also have to develop processes with different requirements and features to enable such models. Quality assurance and quality controls are fundamental parts of the currently accepted GMP standards: manufacturers are expected to adhere to such standards and ensure them in every country where they supply therapies. Regulators such as FDA and EMA routinely inspect manufacturers’ facilities and quality management systems to ensure that patient safety is maintained in every batch that is released in markets. A localized model creates challenges to such approaches as each individual hospital could be considered a manufacturing site, each with their own approaches and facilities out of the management of the originator companies. Regulators may have to inspect hundreds or thousands of individual sites, raising fears that patients may receive therapeutic doses with varying characteristics across different hospitals.

In addition, several post-launch processes may become increasingly difficult: data collection pertaining to real-world use of the therapies; pharmacovigilance processes; product liability assignments; and others. In spite of this, it is worth remembering that distributed manufacturing models are routinely used in the nuclear medicine industry for diagnostic radionuclides such as ^68^Ga and ^99*m*^Tc, which have even shorter half -lives than ^212^Pb and can be produced with generators close to the point of use. It is therefore likely that a regulatory framework can be achieved for an analogous concept in the ART setting.

A centralized model fits within the existing regulatory framework, enabling consistent quality controls across manufacturing sites of a given manufacturer. Such facilities could offer advantages such as improved manufacturing infrastructure for high-volume production, streamlined influx of source material, more uniform rules for developers and better regulatory and quality control. In a centralized model, it should also be easier to assemble and train teams with the relevant manufacturing expertise in this budding new area.

Centralized models do, however, create supply chain risk. A manufacturing network with few facilities and low supply chain redundancy may lead to radionuclide shortages and disrupt patient treatment. For example, in May 2022, Novartis was forced to halt production of both Lutathera and Pluvicto at facilities in Italy, the US and Canada due to quality issues. Delivery of Lutathera was suspended in the US and Canada as a result, and delivery of Pluvicto was also suspended in the US. The disruption led to shortages in Europe and Asia, but these areas were also supplied from another facility in Zaragoza, Spain. Enrolment for clinical trials of Pluvicto stopped globally, as did Lutathera’s clinical studies in the US and Canada (90, 91).

### A way forward: Distributed model

Looking to the future, a middle ground may be the best option in the form of a distributed model, with a moderate number of manufacturing facilities supported by an integrated supply network. This may overcome challenges that prevent rapid scale up on a local level, while addressing challenges such as long patient travel, isotope transport times, supply chain security, and regulatory consistency.

In this model, although not every country (or state in the US) may have its own production and manufacturing facility, multiple sites could ensure that therapies are more accessible, reducing patient travel and therapy transport times. Such a network may also be more resilient to supply chain shocks, and render regulatory compliance more manageable than in the localized model. A network of 10–15 sites per region may be sufficiently redundant for a resilient supply chain and it should be manageable from a regulatory perspective.

Distributed networks are known to be far more stable and productive than centralized alternatives, and the redundancy that would be introduced will be essential for effective and stable therapeutic supply in the future. Taking the internet as example, network redundancy provides multiple paths for traffic, so that data can keep flowing even in the event of a failure. Put simply, more redundancy equals more reliability. The redundancy created by distributed networks can be considered necessary complexity to reduce the probability of failures that could impact the entire network and, ultimately, patients’ lives.

Currently, the unexpected closing of one reactor or one specialized laboratory could already lead to worldwide problems in the supply of medical radionuclides and therapeutics. Other reactors or manufacturing sites may not always absorb the increased demand. This phenomenon was eminently on display during the productions issues of Novartis described above (90, 92).

## Conclusion and future outlook

Alpha radioligand therapeutics (ARTs) offer great promise for the treatment of cancer that is reflected in high expectations for patient impact and financial returns. It is encouraging to see this reflected by the rapid growth of ART-focused companies and expanding clinical pipelines within the field. Future growth will be fueled by further efficacy and safety data from ART clinical trials and real-world results–with expanded investigations of earlier stages of cancer. Thorough investigations of the fundamentals of ART coupled with combination therapies with other modalities, particularly immunotherapeutics, provide fertile ground for academic and industrial researchers alike. Sustained efforts to increase the availability of isotopes by establishing more manufacturing facilities and new methods of production are key to successful growth of the field. Such advances will need to keep pace with each other to avoid situations such as the current expected imbalance between supply and demand of ^225^Ac. Cross-disciplinary training of specialized practitioners to overcome the referral challenges to adoption will also need to be supplemented with an adjustment of financial incentives that puts patients first. New delivery models must also be developed and implemented to provide equal and resilient patient access. This innovation will require that regulatory frameworks evolve at the speed of the rest of the field in order to balance the needs of all stakeholders.

## Author contributions

Both authors listed have made a substantial, direct, and intellectual contribution to the work, and approved it for publication.
